# Science and Engineering Ph.D. Students’ Career Outcomes, by Gender

**DOI:** 10.1371/journal.pone.0133177

**Published:** 2015-08-05

**Authors:** Annamaria Conti, Fabiana Visentin

**Affiliations:** 1 Scheller College of Business and RCEA, Georgia Institute of Technology, Atlanta, Georgia, United States of America; 2 Chair of Economics and Management of Innovation, École Polytechnique Fédérale de Lausanne, Lausanne, Vaud, Switzerland; Stanford University, UNITED STATES

## Abstract

We examine differences in the careers of men and women Ph.D.s from two major European universities. Having performed regression analysis, we find that women are more likely than men to be employed in public administration when the alternatives are either academia or industry. Between the latter two alternatives, women are more likely to be employed in academia. These gender differences persist after accounting for Ph.D.s’ and their supervisors’ characteristics. Gender gaps are smaller for Ph.D.s with large research outputs and for those who conducted applied research. Restricting the analysis to Ph.D.s who pursued postdoc training, women are less likely than men to be employed in highly ranked universities, even after controlling for their research outputs. Finally, we find gender differences in Ph.D.s’ appointment to professorship, which are explained by the Ph.D.s’ publication output and the quality of their postdoc training.

## Introduction

**Fig 1:**
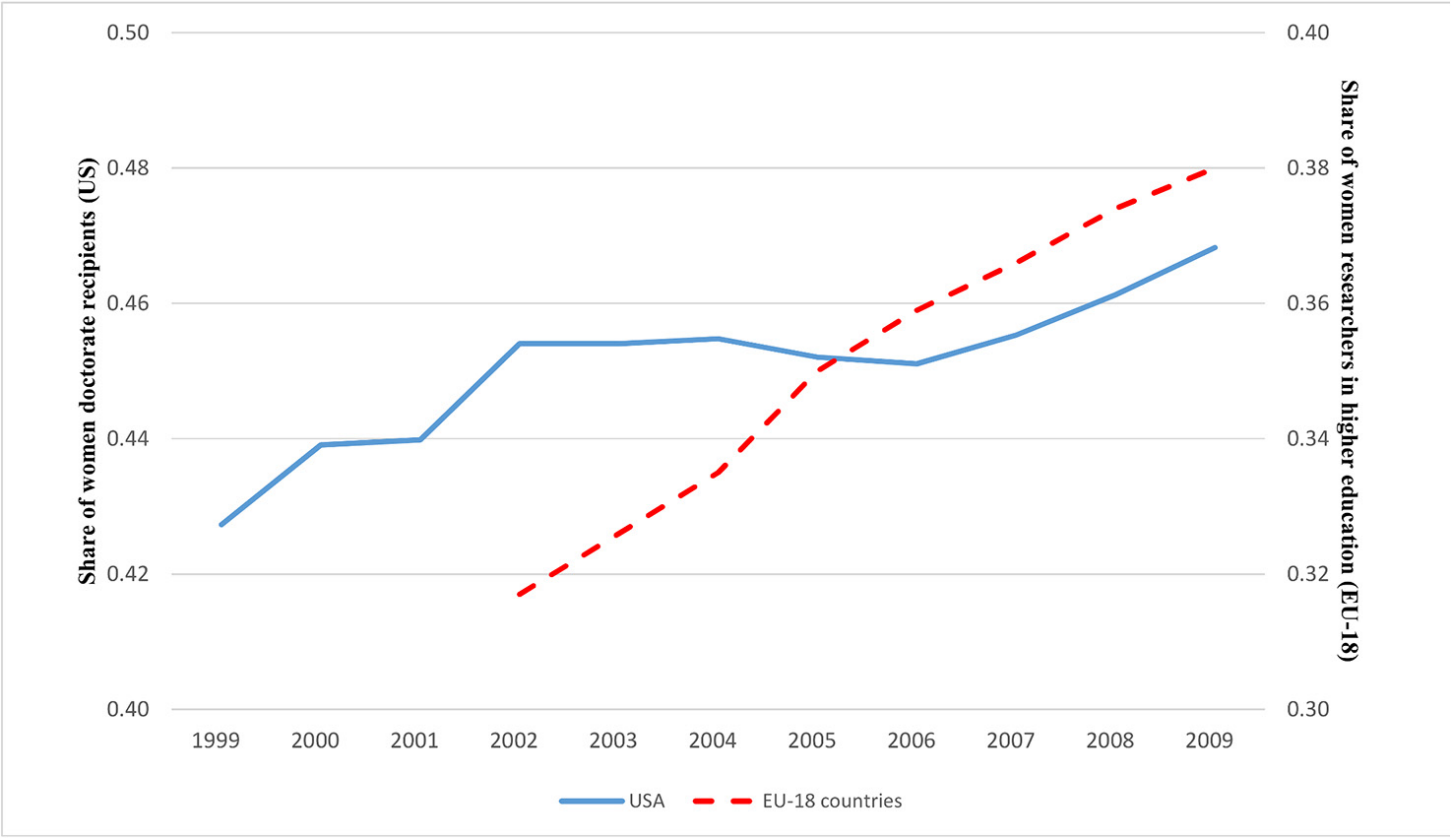
Participation of women in science. Share of women doctorate recipients in the US (solid line) and share of women researchers in the higher education sector in EU-18 countries (dashed line). According to the definition provided by Eurostat, the higher education sector is composed of all universities, colleges of technology, and other institutes of post-secondary education. It also includes research institutes, experimental stations and clinics operating under the direct control of or administered by or associated with higher education establishments. Sources: US National Science Foundation Survey of Earned Doctorates (SED) and Eurostat (Science and Technology Database, Statistics on Research and Development).

In the US and in Europe, the participation of women in science has increased over time (Fig 1). Despite their increased participation, women in the US science context have been found to be less productive [1,2,3], to earn less [4,5], and to receive promotions later than men [6,7]. Moreover, women with postdoc training are more likely than men to find positions in the government and non-profit sectors as opposed to finding employment in academia [8,9]. Little is known, however, about the career patterns of European women in science. With the exception of Mairesse and Pezzoni’s (2015) analysis of French academic women’s promotion patterns, there is a limited understanding of European women’s employment choices after they receive their Ph.D.s [10]. This is an important gap if one considers that Europe is the second-largest producer of highly cited scientific articles, after the US [11]. In addition, in the US and Europe, it is still unclear how the characteristics of Ph.D. and postdoc trainings affect gender differences in career attainments.

To fill these gaps, we use detailed information about Ph.D.s in science and engineering from two major European universities, one located in Sweden and the other in Switzerland. The organization of these universities is very similar to that of other European institutes of technology, including the Polytechnic University of Turin, the Swiss Federal Institute of Technology in Zurich, the Eindhoven University of Technology, and the Karlsruhe Institute of Technology. We have information about Ph.D.s’ employment outcomes after graduation and whether they pursued careers in academia (including research centers), industry, or public administration (including schools and teaching colleges). This is an important distinction in light of the fact that, at least in the US, an increasing number of Ph.D.s are considering careers outside of academia [12,13,14,15].

We find that women are more likely than men to be employed in public administration when the alternatives are either academia or industry. Moreover, they are more likely to be employed in academia than in industry. These differences persist after accounting for their publication outputs during their Ph.D., their involvement in applied projects, and their supervisors’ characteristics. The gender gap in the probability of being employed in public administration disappears when we compare men and women with large publication outputs. The gender gap in the probability of being employed in industry remains when we compare men and women who participated in applied projects during their Ph.D. However, women involved in applied projects are more likely than women not involved in such projects to be employed in industry. When the sample of Ph.D.s who pursued postdoc training is considered, we find that women are less likely than men to be employed after graduation in highly ranked universities, even after controlling for their research outputs. Finally, we find gender differences in Ph.D.s’ appointment to professorship. These differences are explained by the Ph.D.s’ publication output and the quality of their postdoc training.

## Materials and Methods

### Ethics statement

This paper performs secondary analysis of publicly available data and received IRB exemption from the Georgia Institute of Technology. Even though the data is publicly available, we anonymized and de-identified Ph.D. student information in compliance with IRB regulations.

### Sample construction

To build our sample, we obtained complete lists of Ph.D.s who graduated from the Swedish Chalmers University of Technology (Chalmers) and the Swiss Federal Institute of Technology in Lausanne (EPFL) from 1999 to 2009. These institutions share a number of characteristics: they are leading research institutions in their own countries, they focus on science and engineering, and they have extensive collaborations with the industrial sector [15]. Moreover, supervisors directly select Ph.D. applicants at both universities, and Ph.D. duration is fixed to four years [12,16].

From the initial population of Ph.D.s (2,061 at EPFL and 1,290 at Chalmers), we only retained those for whom we had complete information regarding their employment. We gathered this information through extensive searches on the Ph.D.s’ websites, their supervisors’ websites, their publicly available dissertations, and their LinkedIn profiles [12]. The percentage of women we were not able to identify is 35 and the percentage of men is 28. We address possible sample selection concerns in the Results section. The final sample comprises 2,345 students: 1,462 from EPFL and 883 from Chalmers. Women represent 21 percent of the total (20 percent of the EPFL sample and 23 percent of the Chalmers sample).

**Fig 2:**
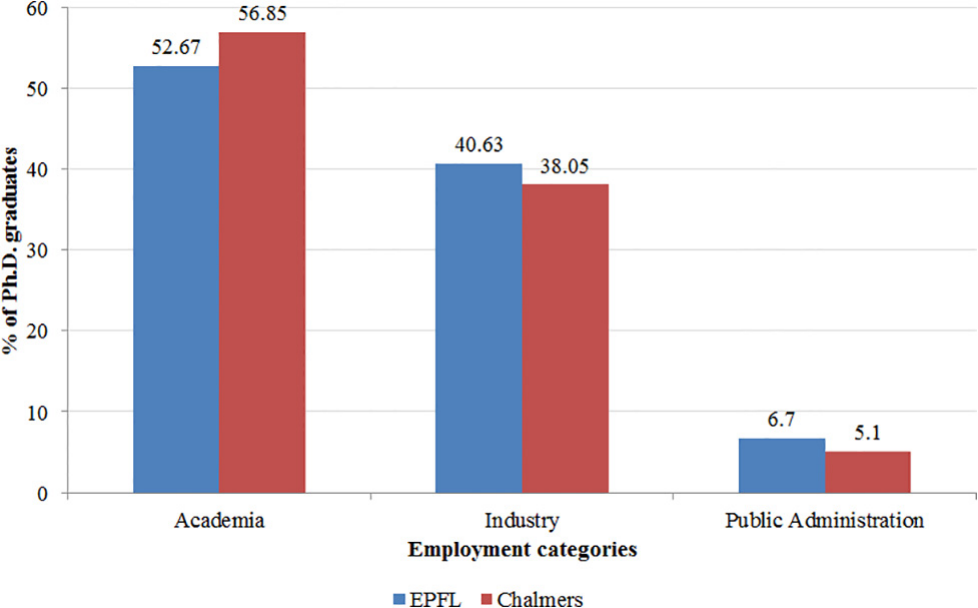
Employment after graduation, by institution. Employment category distribution of EPFL (blue) and Chalmers (red) Ph.D. graduates.

**Fig 3:**
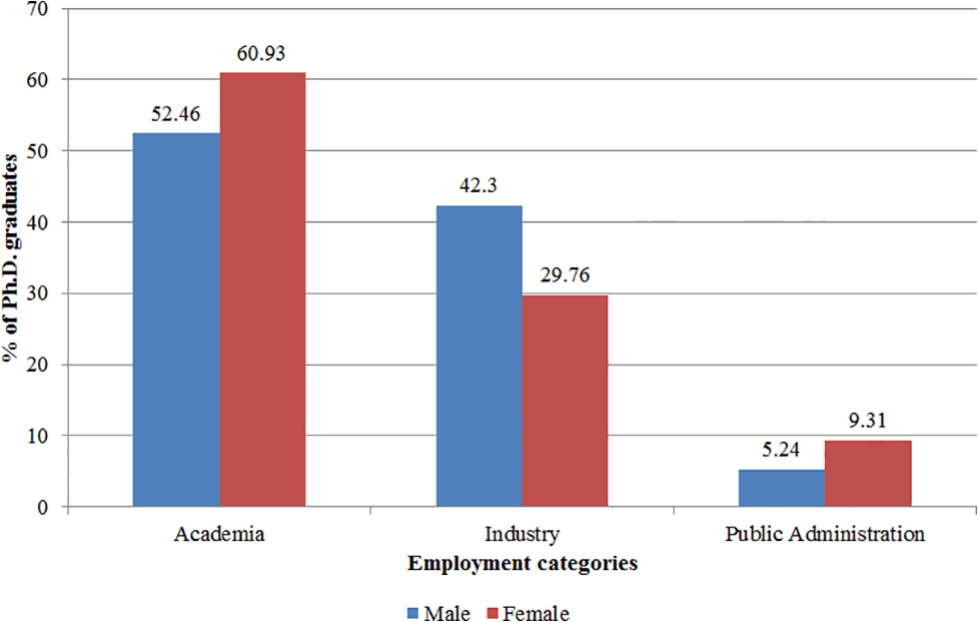
Employment after graduation, by gender. Employment category distribution of male (blue) and female (red) Ph.D. graduates.

Forty-two percent of the Ph.D.s graduated in basic sciences (physics, chemistry, mathematics, and life sciences) and the remaining in engineering. Eighty-four percent of them had only one supervisor assigned. For those with more than one supervisor assigned, we conducted extensive searches to identify their main supervisor [12]. In terms of their career choices after graduation, 1,272 Ph.D.s were employed in academia (1,137 as postdocs, 87 as assistant professors, and 48 as lecturers), 930 were employed in the industrial sector, and 143 were employed in public administration. The majority of Ph.D.s (67 percent) took positions in their graduation country, and we observe no difference in country selection between male and female Ph.D.s. Descriptive statistics reveal that the career patterns of EPFL and Chalmers Ph.D.s are very similar (Fig 2). They also reveal that the percentage of women employed in academia or in public administration is larger than the percentage of men, while the percentage of men is larger in the industry category (Fig 3). Tests for the difference between the means of men and women employed in academia, public administration, and industry reveal that these differences are significant with a p-value of 0.00.

### Statistical methodology

Our initial research questions are: i) Are there gender differences in employment choices of Ph.D.s? and ii) Can such gender differences be explained by observable factors, with a specific focus on the students’ Ph.D. training characteristics? To address these questions, we consider the following employment outcomes after graduation: academia, industry, and public administration. We thus estimate the following multinomial logit model:

$$\text{Pr}\left(y_{i}=j|x_{i}\right)=\frac{\text{exp}\left(x_{i}\beta_{j}\right)}{\sum_{j=1}^{M}\;\text{exp}\left(x_{i}\beta_{j}\right)}$$ (1)

where *j = 1*,*2*,*3*,*…*,*k*,*…*,*M*; Pr(*y*_*i*_ = *j*|*x*_*i*_) is the probability that Ph.D. *i* attains employment category *j*, given ***x***_***i***_; ***x***_***i***_ is a vector of covariates; and **β**_***j***_ is a vector of coefficients pertaining to employment category *j*. In addition to a Ph.D.’s gender, **x**_***i***_ includes demographic variables of age and nationality. We distinguish between domestic and foreign students. Within the category of foreign students, we distinguish between foreign students from EU-15 countries and the remaining foreign students, given that Ph.D.s from outside the EU-15 face considerable limitations in their ability to work in Sweden or Switzerland. We also include an indicator for whether a student had worked prior to starting his or her Ph.D. We measure the research output of a Ph.D. with the number of research articles (including conference proceedings) that a student had published from enrollment to two years after graduation. By so doing, we take into account possible lags between the time a research project is completed and the time its results are published [17]. Moreover, we control for an individual’s involvement in applied projects during the Ph.D. This last measure is an indicator that equals one if a student was granted a patent, had published with industry partners during his or her Ph.D., or was employed by a company during the Ph.D. Details on the variables’ construction and data sources are reported in S1 Table, while descriptive statistics, by gender, are presented in Table 1. Interestingly, men publish more articles than women during their Ph.D. and the difference is statistically significant (p-value = 0.00). Moreover, the percentage of men with prior working experience is significantly larger than the percentage of women (p-value = 0.00).

**Table 1:** Descriptive statistics, by gender.

	Males		Females		
	Mean	SD	Mean	SD	T-test for difference in means
***Ph*.*D*. *demographic and predetermined characteristics***					
Domestic student (reference category)	0.58	0.49	0.49	0.50	***
EU-15 nationality	0.26	0.44	0.31	0.46	**
Non-EU-15 nationality	0.16	0.37	0.20	0.40	**
Age	30.43	2.59	30.54	2.76	
Worked prior to Ph.D.	0.14	0.35	0.08	0.28	***
***Ph*.*D*. *training***					
*Ph*.*D*. *characteristics*					
# of publications during Ph.D.	6.87	6.27	5.54	5.28	***
Involved in applied projects during Ph.D.	0.18	0.38	0.14	0.35	*
*Supervisor characteristics*					
# of publications	32.54	28.52	34.17	30.93	
Had patents granted	0.25	0.43	0.28	0.45	
Involved in EU projects with industrial partners	0.19	0.39	0.18	0.39	

* p<0.10

** p<0.05

*** p<0.01.

1,851 male Ph.D.s; 494 female Ph.D.s. Means, standard errors, and t-tests for the difference in means shown. Supervisor characteristics are measured during the 5 years prior to Ph.D. *i*’s enrollment in the doctoral program.

We include supervisor characteristics because these are likely to be correlated with a Ph.D.’s training. Thus, we control for a supervisor’s publication count in the five years prior to Ph.D. *i*’s enrollment, whether he or she was granted patents during the same period, and was involved in European projects with companies. At both Chalmers and EPFL, European projects are an important component of the total collaborations that professors establish with industrial partners. One possible concern with our strategy is that men and women match with supervisors who have distinct characteristics. Hence, supervisors’ controls may capture Ph.D.s’ unobserved characteristics that are correlated with gender and not the impact of supervisor characteristics on the Ph.D.s.’ career choices. Reassuringly, descriptive statistics presented in Table 1 show that there are not significant differences between men and women Ph.D.s with respect to the supervisor characteristics being examined. Moreover, logit regression estimates show that these differences remain insignificant even when we account for the other Ph.D. characteristics (S2 Table).

We add graduation-year fixed effects, university-research field fixed effects (engineering, life sciences, chemistry, physics, and mathematics), and measures for labor market conditions at graduation. As a measure for labor market supply, we use the size of Ph.D. *i*’s graduation cohort, which is defined as the count of students who graduated in year *t* in the same field as *i* [12]. The fields we consider are basic sciences and engineering. As in Conti and Visentin (2014), we include in the count of students only those Ph.D.s who are potential competitors to Ph.D. *i* in the job market [12]. In the case of EPFL, these are Ph.D.s who had graduated from EPFL and from the Swiss Federal Institute of Technology in Zurich. In the case of Chalmers, potential competitors are Ph.D.s from Chalmers and from the Swedish Royal Institute of Technology, which offers very similar doctoral programs to Chalmers. As a measure for labor market demand, we include an indicator that increases in value with higher graduation country GDP growth. We also control for the availability of positions in R&D-intensive companies with the number of patent applications filed by Sweden and Switzerland at the European Patent Office in the year in which a Ph.D. graduated. We proxy the availability of postdoctoral positions at a Ph.D.’s university of graduation with the number of professors affiliated with EPFL and Chalmers in the same field as Ph.D. *i*. Additionally, we control for the availability of postdoc positions in the US, distinguishing between basic sciences and engineering, since many Ph.D.s pursue postdoc training in the US. This measure is defined as the difference between the number of postdocs hired by US universities in a given year and the number of US Ph.D.s who graduated in that year in the same field as Ph.D. *i*.

In the second part of the analysis, we restrict our attention to those 1,137 Ph.D.s who pursued, after their graduation, a postdoc training and address the following two questions: i) Are there gender differences in the Ph.D.s’ likelihood of attaining positions in highly ranked universities (including research institutions)? ii) Are there gender differences in the hazard of appointment to professorship? By appointment to professorship we mean appointment to a position of assistant professor, which encompasses research as well as teaching duties. We thus exclude senior lecturers from the category of assistant professors because they tend to focus solely on teaching.

**Fig 4:**
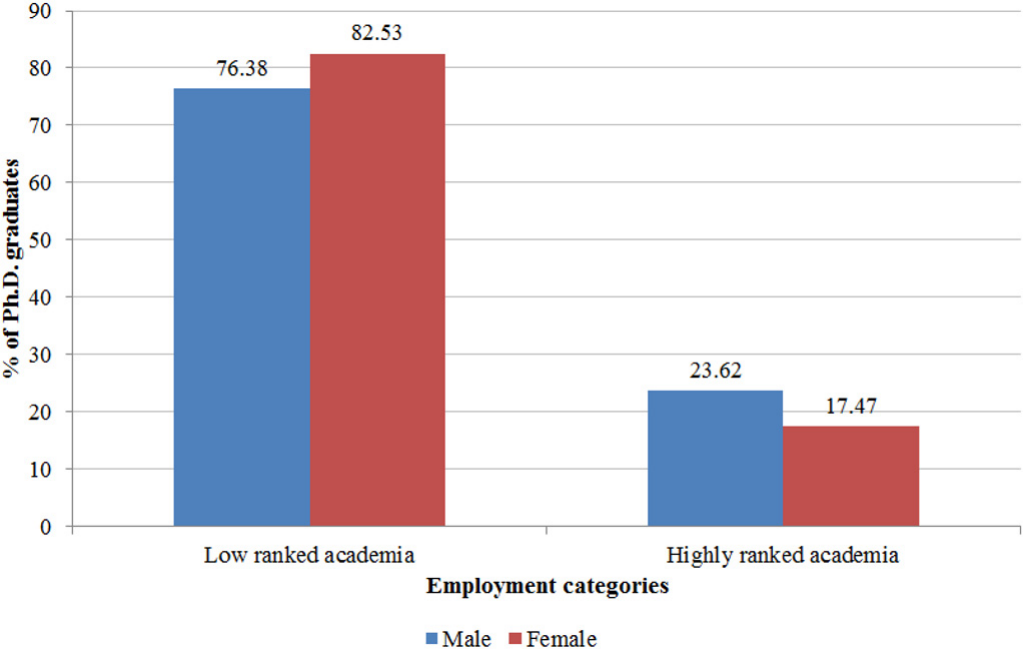
Postdoc training after graduation, by gender. Employment category distribution of male (blue) and female (red) Ph.D. graduates who pursued postdoc training. Universities are classified as highly ranked if they are in the top quartile for the number of articles published in the same field as Ph.D. *i* and if they are located outside of *i*’s graduation country.

To answer the first question, we estimate a logit model for the probability that Ph.D. *i* is enrolled as a postdoc in a highly ranked university:

$$\text{Pr}\left(y_{i}=1|x_{i}\right)=\frac{\text{exp}\left(x_{i}\beta \right)}{1+\text{exp}\left(x_{i}\beta \right)}$$ (2)

where Pr(*y*_*i*_ = 1|*x*_*i*_) is the probability that Ph.D. *i* received a postdoc training in a highly ranked university, given ***x***_***i***_; ***x***_***i***_ is the same vector of covariates used in Eq (1); and ***β*** is a vector of coefficients. We classify universities as highly ranked if they are in the top quartile for the number of publications published in the same field as Ph.D. *i*. At both Chalmers and EPFL, Ph.D.s who intend to pursue careers in academia are strongly encouraged to take postdoc positions abroad, especially in the US. Thus, we consider postdocs in Sweden or Switzerland to be low ranked. According to our categorization, 24 percent of male postdocs and 18 percent of female postdocs took positions in highly ranked universities (Fig 4) and the gender difference is significant with a p-value of 0.03. The results we present in this paper are robust to alternative classification criteria in which, for instance, we classify as postdoc positions in highly ranked universities the ones at Chalmers and EPFL, provided that the concerned Ph.D.s had moved to a different research group than their supervisor’s [12].

To address the question of whether there are gender differences in the hazard of appointment to professorship, we estimate a competing-risks model that accounts for the fact that a Ph.D. is at risk of transitioning to multiple different occupations, in addition to being appointed assistant professor. The hazard of becoming assistant professor, h_j_(t|**x**_**i**_), is modeled as:

$$h_{i}\left(t|x_{i}\right)=h_{j,0}\left(t\right)\text{exp}\left(x_{i}\beta_{x}\right)$$ (3)

where *h*_*j*,0_(*t*) is the baseline hazard; ***x***_***i***_ is a vector that contains the same covariates as in Eq (1) plus characteristics of a Ph.D.’s postdoc training (publication count and whether the hosting university is highly ranked) and labor market conditions at time *t*; and **β**_*x*_ is a vector of coefficients. In the case of GDP growth at time *t*, we compute the average between the GDP growth of the country in which Ph.D. *i* was working in *t-1* and the GDP growth of the country in which Ph.D. *i* was working at time *t*. The results from estimating Eq (1)–(3) are reported in the next section.

## Results

### Ph.D. employment career choices

We begin by estimating Eq (1). We initially consider as employment alternatives positions in academia and positions outside of academia. The corresponding results are displayed in Table 2. We then categorize positions outside of academia into positions in industry and public administration. The results from this specification are reported in Table 3.

**Table 2:** Logit regression results for being employed in academia, after graduation.

Base outcome: Employment outside of academia			
	I	II	III
Female	1.242*	1.409***	1.406***
	(0.140)	(0.160)	(0.158)
*Ph*.*D*. *demographic and predetermined characteristics*			
EU-15 nationality	1.287**	1.233*	1.186
	(0.141)	(0.139)	(0.135)
Non-EU-15 nationality	2.049***	1.984***	1.847***
	(0.280)	(0.274)	(0.259)
Age	0.995	1.022	1.037**
	(0.017)	(0.018)	(0.018)
Worked prior to Ph.D.	0.428***	0.425***	0.504***
	(0.063)	(0.064)	(0.076)
*Ph*.*D*. *publication output*			
# of publications during Ph.D.		1.877***	1.976***
		(0.119)	(0.127)
*Ph*.*D*. *involvement in applied projects*			
Involved in applied projects during Ph.D.			0.344***
			(0.044)
*Supervisor publication output*			
# of publications		0.921	0.925
		(0.052)	(0.055)
*Supervisor involvement in applied projects*			
Had patents granted			0.989
			(0.122)
Involved in EU projects with industrial partners			1.121
			(0.131)
Pseudo R2	0.05	0.09	0.12

* p<0.1

** p<0.05

*** p<0.01

Coefficients are odds ratios. N = 2,345. Standard errors clustered around supervisors are in parentheses. Controls include labor market characteristics at graduation, university-research field fixed effects, and graduation-year fixed effects.

**Table 3:** Multinomial logit regression results for Ph.D. career choices.

Base outcome: Academia	Industry			Public Administration		
	I	II	III	IV	V	VI
Female	0.690***	0.609***	0.606***	1.662***	1.464**	1.471**
	(0.086)	(0.076)	(0.075)	(0.322)	(0.284)	(0.283)
*Ph*.*D*. *demographic and predetermined characteristics*						
EU-15 nationality	0.808*	0.841	0.878	0.609**	0.643*	0.662*
	(0.091)	(0.097)	(0.102)	(0.140)	(0.150)	(0.155)
Non-EU-15 nationality	0.511***	0.528***	0.572***	0.357***	0.365***	0.376***
	(0.074)	(0.078)	(0.086)	(0.106)	(0.110)	(0.113)
Age	0.987	0.961**	0.946***	1.103***	1.067**	1.057*
	(0.019)	(0.018)	(0.018)	(0.034)	(0.034)	(0.034)
Worked prior to Ph.D.	2.614***	2.625***	2.198***	0.971	0.962	0.900
	(0.394)	(0.398)	(0.334)	(0.297)	(0.302)	(0.288)
*Ph*.*D*. *publication output*						
# of publications during Ph.D.		0.534***	0.505***		0.522***	0.507***
		(0.036)	(0.034)		(0.061)	(0.060)
*Ph*.*D*. *involvement in applied projects*						
Involved in applied projects during Ph.D.			3.121***			1.783**
			(0.413)			(0.499)
*Supervisor publication output*						
# of publications		1.104	1.098		0.979	0.988
		(0.067)	(0.069)		(0.107)	(0.112)
*Supervisor involvement in applied projects*						
Had patents granted			1.059			0.751
			(0.138)			(0.175)
Involved in EU projects with industrial partners			0.840			1.241
			(0.104)			(0.269)

* p<0.1

** p<0.05

*** p<0.01.

Coefficients are relative risk ratios. N = 2,345. Standard errors clustered around supervisors are in parentheses. Controls include labor market characteristics at graduation, university-research field fixed effects, and graduation-year fixed effects.

In Table 2, displayed coefficients are odds ratios. Ratios greater than one indicate that an increase in the regressor leads to a higher probability that an outcome *j* will occur rather than the base outcome, with the opposite being true for ratios less than one. Standard errors are clustered around Ph.D. supervisors. Employment outside of academia is the base outcome. We gradually introduce the aforementioned controls to assess whether and how these controls affect gender differences in Ph.D.s’ employment attainments. Column I includes the gender indicator, Ph.D.s’ demographic and predetermined characteristics, measures for labor market conditions at graduation, and fixed effects. As shown, women are more likely than men to be employed in academia, the coefficient of the female dummy being significant at the 10 percent confidence level.

As reported in Table 1, women publish fewer scientific articles than men. We expect Ph.D. research output to be a source of negative correlation between being female and the probability of working in academia after graduation. In column II, we include both a Ph.D.’s research output and the one of the supervisor with which the Ph.D. is associated. Not surprisingly, Ph.D. research output is significantly and positively correlated with the probability that a student is employed in academia, following graduation. In line with our expectations, the magnitude of the female dummy coefficient increases, relative to the corresponding result in column I, and the coefficient’s significance improves to the 1 percent confidence level.

It could be that female Ph.D.s are more likely to work in academia because they are less involved in applied projects during their training. We explore this possibility in column III, where we control for whether Ph.D.s participated in applied projects. We also include indicators for whether a supervisor was granted patents and collaborated with firms during his or her appointment. The indicator describing a Ph.D.’s involvement in applied projects is a significant, negative predictor of a Ph.D.’s probability of being employed in academia. With the inclusion of this control, the female dummy coefficient decreases slightly, as expected, but remains highly significant.

In Table 3, we further explore the results obtained so far and compare employment in academia to employment in industry and employment in public administration. Reported coefficients are relative risk ratios. Ratios greater than one indicate that an increase in the regressor leads to a higher probability that an outcome *j* will occur rather than the base outcome, with the opposite being true for ratios less than one. Standard errors are clustered around Ph.D. supervisors. Employment in academia is the base outcome. Regression specifications in columns I and IV include the gender indicator, Ph.D.s’ demographic and predetermined characteristics, measures for labor market conditions at graduation, and fixed effects. The results indicate that women are significantly more likely than men to work in public administration than academia or industry. Moreover, they are more likely to work in academia than they are in industry.

One reason why women are more likely to be employed in public administration could be that they are less productive. However, upon introducing a Ph.D.s’ research output and the output of his or her supervisor (columns II and V), women remain more likely than men to work in public administration.

Women could be less likely to work in industry relative to academia and public administration because they are less involved in applied projects. Having controlled for whether Ph.D.s participated in applied projects during their training and for whether their supervisors were granted patents and had collaborated with firms, the gender gap in industry employment remains statistically significant (columns III and VI).

One possible concern with the estimates presented in Tables 2 and 3 is that they may be inconsistent due to sample selection bias. Results provided in S3 Table show that being female is negatively correlated with the probability of being included in our sample, although the significance of this correlation declines once we control for a number of characteristics available for both in and out-of-sample PhD.s. To address the possibility that the results in Tables 2 and 3 may be inconsistent, we estimate two-step Heckman selection models [18] for the probability that i) a Ph.D. is employed in academia, ii) he or she is employed in public administration, and iii) he or she is employed in industry (S3 and S4 Tables). In the selection equation, the probability of being included in the sample is modeled as a function of whether a Ph.D.’s last name appears more than once in our sample, the logic being that it is difficult to identify biographical information for Ph.D.s with a common last name. This variable is unlikely to be correlated with the Ph.D.s’ career choices, thus providing a foundation to the validity of the exclusion restriction imposed. In an alternative specification, we substitute the dummy for whether a Ph.D. had a common last name with the number of times a Ph.D.’s last name appeared in our database [19]. We also control for a Ph.D.’s nationality, age, publication output, gender, measures for labor market conditions at graduation, graduation-year and university-research field fixed effects. In the career outcome equations, we include all the controls listed in Eq (1) and the Mills’ ratio obtained from the selection equation. Standard errors are clustered around Ph.D.s from the same university-research field and graduation year. Reassuringly, regardless of the employment category examined, the Mills’ ratio is insignificantly different from zero, suggesting that the selection equation and the employment outcome equations could be treated as independent. Importantly, the coefficients of the female indicator, once we include the Mills’ ratio, are very similar to those without including the Mills’ ratio, thus supporting the validity of the estimates in Tables 2 and 3.

As an extension to the findings presented in Table 3, we examine how the gender gap in employment attainments varies when we compare men and women with similar research profiles. The results are displayed in Table 4. One of the results we found is that women are more likely than men to work in public administration. In Panel A of Table 4, we assess whether this gender gap persists among those Ph.D.s with a large number of publications. Coefficients are odds ratios and standard errors are clustered around supervisors. The base outcome is employment outside of public administration. For ease of interpretation, we substitute the publication count variable with an indicator for whether a Ph.D. had published more than his or her field’s median number of publications. We then interact this indicator with the gender variable. Women with a large publication output appear to be significantly less likely to be employed in public administration than women without such an output (p-value = 0.00). A test of the difference between the impact of women with a large publication output and the one of men with the same output on the probability of working in public administration fails to reject the null hypothesis that the difference is zero (p-value = 0.57). Overall, our results indicate that there is no gender gap among Ph.D.s with high levels of research output. However, there is a very large gender gap among Ph.D.s with low levels of research output.

**Table 4:** Logit regression results for Ph.D. career choices.

	Odds ratios
**PANEL A:** Probability of being employed in public administration	
Female	2.399***
	(0.502)
Large publication output	0.748
	(0.163)
Large publication output * Female	0.323**
	(0.159)
Other controls	✓
Pseudo R2	0.07
**PANEL B:** Probability of being employed in industry	
Female	0.577***
	(0.080)
Ph.D. involvement in applied projects	2.927***
	(0.424)
Ph.D. involvement in applied projects*Female	1.012
	(0.331)
Other controls	✓
Pseudo R2	0.12

** p<0.05

*** p<0.01.

Coefficients are odds ratios. N = 2,345. Standard errors clustered around supervisors are in parentheses. Controls include Ph.D. demographic and predetermined characteristics, controls for a Ph.D.’s training (including supervisor characteristics), labor market characteristics at graduation, university-research field fixed effects, and graduation-year fixed effects.

In Panel B, we explore whether there are gender differences in the probability of working in industry relative to working academia or public administration, among Ph.D.s involved in applied projects. We use the same statistical model as in Panel A. We interact the gender variable with the indicator for whether Ph.D.s pursued applied projects. The base outcome is employment outside of industry. A test of the difference between the impact of women involved in applied projects and the impact of men involved in the same projects on the probability of working in industry rejects the null hypothesis that the difference is equal to zero (p-value = 0.06). However, women involved in applied projects are more likely than women not involved in such projects to work in industry (p-value = 0.00).

### Ph.D. employment career choices: Ph.D.s who pursued postdoc positions

Next, we take a closer look at those Ph.D.s who pursued postdoc training. We ask whether there are gender differences in their likelihood of pursuing postdoc positions in highly ranked universities after graduation (Eq (2)) and in their hazard of appointment to professorship (Eq (3)).

The results from estimating Eq (2) are reported in Table 5. We use the same controls as those employed in Tables 2 and 3. As shown, women appear to be less likely than men to take positions in highly ranked institutions after graduation, even after controlling for the number of articles published during their Ph.D. training and their supervisor’s research output.

**Table 5:** Logit regression results for the probability of pursuing a postdoc training in a highly ranked university, after graduation.

	Odds ratio
Female	0.673*
	(0.140)
Other controls	✓
Pseudo R2	0.09

* p<0.10.

We restrict the sample to Ph.D.s who pursued postdoc training after graduation. N = 1,137. The coefficient is an odds ratio. Standard errors clustered around supervisors are in parentheses. Controls include Ph.D. demographic and predetermined characteristics, Ph.D. number of publications and involvement in applied projects, supervisor characteristics, labor market characteristics at graduation, university-research field fixed effects, and graduation-year fixed effects.

**Fig 5:**
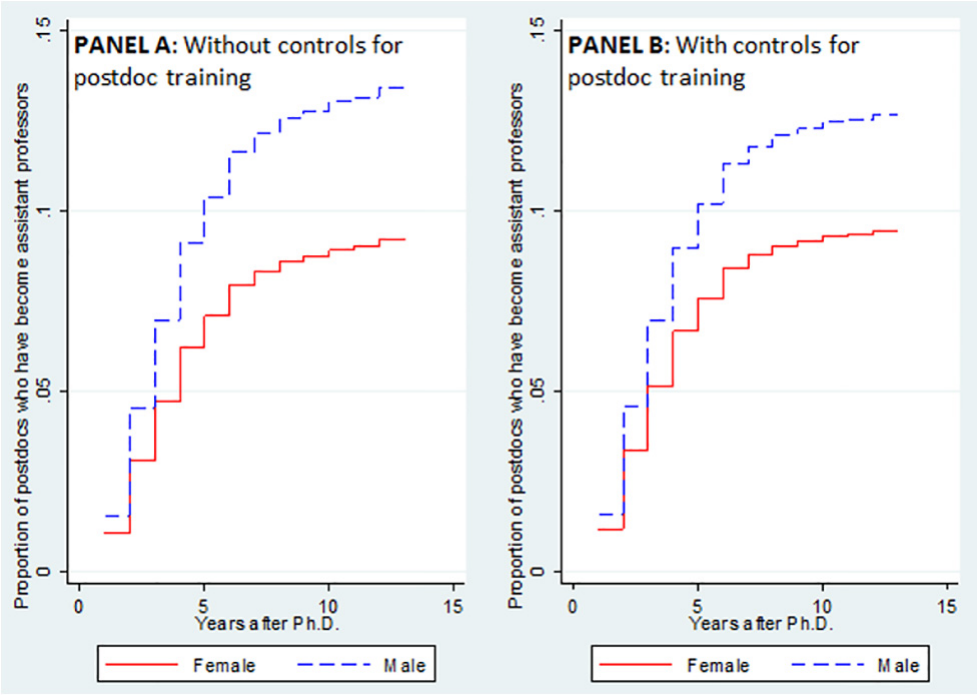
Cumulative incidence functions for appointment to professorship. We report the proportions of male (dashed line) and female (solid line) postdocs who have become assistant professors. The cumulative incidence functions represented in Panel A are derived by estimating Eq (3). We control for Ph.D. demographic and predetermined characteristics, Ph.D. number of publications and involvement in applied projects, supervisor characteristics, labor market characteristics at graduation and at time *t*, university-research field fixed effects, and graduation-year fixed effects. The cumulative incidence functions represented in Panel B differ from those in Panel A in that we control for the characteristics of a Ph.D.’s postdoc training.

Fig 5 plots the cumulative incidence functions derived from estimating Eq (3). These functions give the proportion of Ph.D.s at time *t* who have become assistant professors, but could have transitioned into any other occupation. Standard errors are clustered around Ph.D.s. In Panel A, we exclude from the covariates in Eq (3) the characteristics of a Ph.D.’s postdoc training, namely the yearly number of articles published and the rank of the postdoc university. The difference between men (dashed line) and women (solid line) in appointment to professorship is large and statistically significant (p-value = 0.05). In Panel B, we include the characteristics of a Ph.D.’s postdoc training and, this time, the gender gap is no longer significant at conventional levels. However, given the p-value of 0.14, we cannot completely rule out the existence of a gender gap in a Ph.D.’s appointment to professorship.

## Discussion

We show that gender gaps exist in the employment outcomes for Ph.D.s in Europe. This finding is surprising in light of the organizational differences, including gender policies, between European and US universities. Our findings could be explained by the fact that women face obstacles in accessing certain employment categories, i.e. highly-ranked foreign universities and industry, or by the fact that women deliberately choose to avoid these careers. Provided that women face obstacles in accessing certain employment categories, then policy makers could implement *ad hoc* measures to reduce gender gaps. For instance, we found that women are less likely than men to be appointed professors and one important driver is the fact that women are less likely to undergo postdoc training in highly ranked foreign universities. If women elect to pursue a career as professors in European academia, then grants aimed at encouraging women’s mobility could reduce the gender gap in appointment to professorship. We also found that women are less likely than men to be employed in industry and that working in applied projects during a Ph.D. program is an important predictor of employment in industry after graduation. Once again, if working in industry is a high priority for women, then policy makers and university administrators could encourage them to participate in applied research projects during their Ph.D to improve the odds of their working in industry. In parallel, policy makers could set incentives for firms to hire female Ph.D.s who participate in those projects.

This study is not without limitations, which open venues for future research. For instance, we applied our analysis to two major European universities that share several traits with other institutes of technology. Future research could assess the gender gap in the context of universities that are less focused on science and engineering disciplines. Moreover, although we can control for a large number of Ph.D. characteristics, we lack information about the marital status of women and whether they have children [20]. It is possible that the gender gap we found is even larger for married women and for those with children. While the Ph.D. predetermined characteristics that we control for allow us to mitigate the problem of selection into a given research group, future research could validate our analysis using *ad hoc* econometric models. Another limitation is that some of our controls are imperfectly measured. For instance, we proxied the Ph.D.s’ research output with the number of their research articles, without controlling for article quality. Our choice was dictated by the fact that standard measures of publication quality, i.e. journal impact factors and citations, are not available for all publication types [16]. Controlling for the Ph.D.s’ research quality may reduce the gender gap in Ph.D.s’ career outcomes. Additionally, given the nature of our data, we do not observe unemployment periods and, more generally, periods during which Ph.D.s leave the job market. Reassuringly, discussions with administrators from both universities revealed that these periods usually last less than six months. Finally, while our study represents an important contribution to the extant literature in that it goes beyond examining the careers of women in academia, future research could distinguish between industry employments that are more or less close to academic organization and values [21].

## Supporting Information

S1 Table Details on the variables’ construction and data sources. (DOCX)

S2 Table Logit regression estimates for the probability that a Ph.D. student is female. Coefficients are odds ratios. Ratios greater than one indicate that an increase in the regressor leads to a higher probability that a Ph.D. student is female, with the opposite being true for ratios less than one. Standard errors clustered around supervisors are in parentheses. Supervisor characteristics are measured during the 5 years prior to Ph.D. *i*’s enrollment in the doctoral program. Controls include Ph.D. demographic and predetermined characteristics, Ph.D. number of publications and involvement in applied projects, labor market characteristics at graduation, university-research field fixed effects, and graduation-year fixed effects.
(DOCX)

S3 Table Probit regression estimates for the probability that a Ph.D. is included in the sample. Coefficients are average marginal effects. Standard errors clustered around students from the same university-research field and graduation-year are in parentheses. *** p<0.01. Controls include labor market characteristics at graduation, university-research field fixed effects, and graduation-year fixed effects.
(DOCX)

S4 Table Linear probability models for being employed in academia, industry, and public administration (with Mills’ ratios included). Standard errors clustered around students from the same university-research field and graduation-year are in parentheses. ** p<0.05, *** p<0.01. Controls include Ph.D. demographic and predetermined characteristics, Ph.D. number of publications and involvement in applied projects, supervisor characteristics, labor market characteristics at graduation, university-research field fixed effects, and graduation-year fixed effects. We report coefficient estimates with and without the Mills’ ratio.
(DOCX)
