# Supplementary material for: Science and Engineering Ph.D. Students’ Career Outcomes, by Gender
Source: PLoS One. 2015 Aug 5;10(8):e0133177. doi: 10.1371/journal.pone.0133177 (PMC4526637; doi:10.1371/journal.pone.0133177)
Supplement: S1 Table — (DOCX) [file pone.0133177.s001.docx]

**S1 Table. Details on the variables’ construction and data sources**

| **Variable** | **Definition** | **Source** |
| --- | --- | --- |
| ***Ph.D. employment outcomes*** | | |
| We considered the first Ph.D. employment outcome after graduation. We classify employment in three exaustive and mutually exclusive categories: academia, industry, and public administration*, ** | | |
| Academia | Dummy variable equal to 1 if a Ph.D. obtained a position in a university (including public research centers) | Ph.D.s' CVs (including those reported in the Ph.D.s' dissertations) |
| Industry | Dummy variable equal to 1 if a Ph.D. obtained a position in industry | Ph.D.s' CVs (including those reported in the Ph.D.s' dissertations) |
| Public administration | Dummy variable equal to 1 if a Ph.D. obtained a position in public administration (including schools and teaching colleges) | Ph.D.s' CVs (including those reported in the Ph.D.s' dissertations) |
| ***Ph.D. demographic and predetermined characteristics*** | | |
| Female | Dummy variable equal to 1 if a Ph.D. is female | Ph.D.s' CVs (including those reported in the Ph.D.s' dissertations) |
| Domestic student (reference category) | Dummy variable equal to 1 if a Ph.D. obtained his or her master's degree in Switzerland (for EPFL Ph.D.s) or in Sweden (for Chalmers Ph.D.s) | Ph.D.s' CVs (including those reported in the Ph.D.s' dissertations) |
| EU-15 nationality | Dummy variable equal to 1 if a Ph.D. obtained his or her master's degree from a EU-15 country | Ph.D.s' CVs (including those reported in the Ph.D.s' dissertations) |
| Non-EU-15 nationality | Dummy variable equal to 1 if a Ph.D. obtained his or her master's degree from a foreign country that is not part of the EU-15 | Ph.D.s' CVs (including those reported in the Ph.D.s' dissertations) |
| Age | Ph.D. age at graduation | Ph.D.s' CVs (including those reported in the Ph.D.s' dissertations) |
| Worked prior to Ph.D. | Dummy variable equal to 1 if a Ph.D. had a work experience previous to his or her entry in the Ph.D. program. We do not consider job positions lasting fewer than 6 months | Ph.D.s' CVs (including those reported in the Ph.D.s' dissertations) |
| ***Ph.D. training*** | | |
| Number of publications during Ph.D. | Number of research articles (including conference proceedings) published from the moment a Ph.D. is enrolled in a doctoral program until two years after graduation. When Ph.D.s had common last names, we assigned them publications using information about their affiliation and their supervisors' names, which we gleaned from CV data | Scopus |
| Large publication output | Dummy variable equal to 1 if the number of a Ph.D.'s publications is greater than the field's median. The fields we consider are basic science and engineering. The field's median is computed using the number of publications of all Ph.D.s who graduated in the same year as Ph.D. *i,* in his or her field | Scopus |
| Involved in applied projects during Ph.D. | Dummy variable equal to 1 if a Ph.D. was granted at least one patent, had published articles with industrial partners, or had worked with a company during her Ph.D. | Thomson Reuters, Scopus, Ph.D.s' CVs |
| ***Supervisor characteristics*** | | |
| Number of publications | Number of research articles (including conference proceedings) that a Ph.D. i's supervisor published in the 5 years prior to Ph.D. *i*'s enrollment in the Ph.D. program | Scopus |
| Had patents granted | Dummy variable equal to 1 if a supervisor was granted at least one US patent in the 5 years prior to Ph.D. *i*'s enrollment in the Ph.D. program | Thomson Reuters |
| Involved in EU projects with industrial partners | Dummy variable equal to 1 if a supervisor was involved in European projects with industrial partners in the 5 years prior to Ph.D. *i*'s enrollment in the Ph.D. program | European Commission CORDIS website |
| ***Postdoc training*** | | |
| Yearly number of publications | Number of yearly research articles (including conference proceedings) published after two years Ph.D. *i* completed his or her Ph.D. | Scopus |
| Highly ranked university | Dummy variable equal to 1 if a Ph.D.'s postdoc university is in the top quartile for the number of publications published, during the last decade, in the same field as Ph.D. *i.* We consider the following fields: physics, mathematics, chemistry, material science, and engineering | Scopus |
| ***Labor market characteristics at graduation*** | | |
| Ph.D. cohort size | Number of Ph.D.s who graduated in the same year and in the same field as Ph.D. *i*. The fields we consider are basic science and engineering. These students were enrolled with either EPFL or ETH, if *i* is from EPFL, and with Chalmers or KTH, if *i* is from Chalmers | Universities' websites and countries' Statistical Offices |
| GDP growth, at graduation | Categorical variable assuming values that increase with the GDP growth of Ph.D. *i*'s graduation country, with 0 indicating a recession period and 3 indicating an high-growth period. For Sweden, the variable is =0 in the years 2008-2009, =1 in 2001, =2 in 2002, 2003, 2005, and 2007, =3 in 1999, 2000, 2004, and 2006. For Switzerland, the variable is =0 in 2009, =1 in 2002 and 2003, =2 in 1999, 2001, 2004, 2005, and 2008, =3 in 2000, 2006, and 2007 | World Bank |
| Number of professors in graduation country | Number of professors affiliated with EPFL or Chalmers, during Ph.D. *i*'s graduation year, for the field in which *i* is specialized, i.e. engineering and basic sciences | Universities' websites and countries' Statistical Offices |
| Number of graduation country's EPO patent applications | Number of patent applications that Sweden and Switzerland had filed at the European Patent Office during Ph.D. *i*'s graduation year | OECD |
| US postdoc positions | Difference between the number of postdocs hired by US universities in a given year and the number of US Ph.D.s who graduated in the same year and field as a Ph.D. *i.* The fields we consider are basic science and engineering | National Science Foundation: http://www.nsf.gov/statistics/nsf13331/pdf/tab28.pdf and http://www.nsf.gov/statistics/nsf13331/pdf/tab29.pdf |
| ***Labor market characteristics at time t****** | | |
| GDP growth at time *t* | Average between the GDP growth of the country in which Ph.D. *i* worked in *t-1* and the GDP growth of the country in which Ph.D. *i* worked at time *t* | World Bank |
| * We only considered those CVs that contained complete information about the Ph.D.s' employment. We removed from the sample those Ph.D.s for which we found gaps longer than one year in their employment history. | | |
| ** In most European countries, when freshly graduated Ph.D.s take research positions in academia other than assistant professor positions, the terms “postdoc” and “research scientist” are almost used interchangeably. Hence, and unfortunately, we cannot distinguish between postdocs and research scientists, in the year after graduation. | | |
| *** In "Labor market characteristics at time *t*" we also include the variables listed under "Labor market characteristics at graduation" and measured at time *t*. | | |
