# Supplementary material for: Science and Engineering Ph.D. Students’ Career Outcomes, by Gender
Source: PLoS One. 2015 Aug 5;10(8):e0133177. doi: 10.1371/journal.pone.0133177 (PMC4526637; doi:10.1371/journal.pone.0133177)
Supplement: S2 Table — Coefficients are odds ratios. Ratios greater than one indicate that an increase in the regressor leads to a higher probability that a Ph.D. student is female, with the opposite being true for ratios less than one. Standard errors clustered around supervisors are in parentheses. Supervisor characteristics are measured during the 5 years prior to Ph.D. i’s enrollment in the doctoral program. Controls include Ph.D. demographic and predetermined characteristics, Ph.D. number of publications and involvement in applied projects, labor market characteristics at graduation, university-research field fixed effects, and graduation-year fixed effects. (DOCX) [file pone.0133177.s002.docx]

**S2 Table. Logit regression estimates for the probability that a Ph.D. student is female**

|  | Odds ratios |
| --- | --- |
|  | |
| *Supervisor characteristics* |  |
| # of publications | 1.061 |
|  | (0.077) |
| Had patents granted | 1.104 |
|  | (0.088) |
| Involved in EU projects with industrial partners | 0.953 |
|  | (0.138) |
| Other controls | ✓ |
| Pseudo R2 | 0.06 |
| N obs | 2345 |

Coefficients are odds ratios. Ratios greater than one indicate that an increase in the regressor leads to a higher probability that a Ph.D. student is female, with the opposite being true for ratios less than one. Standard errors clustered around supervisors are in parentheses. Supervisor characteristics are measured during the 5 years prior to Ph.D. *i*’s enrollment in the doctoral program. Controls include Ph.D. demographic and predetermined characteristics, Ph.D. number of publications and involvement in applied projects, labor market characteristics at graduation, university-research field fixed effects, and graduation-year fixed effects.
