# Supplementary material for: Science and Engineering Ph.D. Students’ Career Outcomes, by Gender
Source: PLoS One. 2015 Aug 5;10(8):e0133177. doi: 10.1371/journal.pone.0133177 (PMC4526637; doi:10.1371/journal.pone.0133177)
Supplement: S3 Table — Coefficients are average marginal effects. Standard errors clustered around students from the same university-research field and graduation-year are in parentheses. *** p<0.01. Controls include labor market characteristics at graduation, university-research field fixed effects, and graduation-year fixed effects. (DOCX) [file pone.0133177.s003.docx]

**S3 Table. Probit regression estimates for the probability that a Ph.D. is included in the sample**

|  | Average Marginal Effects |
| --- | --- |
|  | |
| ***Ph.D. demographic and predetermined characteristics*** | |
| Female | -0.046*** |
|  | (0.018) |
| EU-15 nationality | -0.157*** |
|  | (0.030) |
| Non-EU-15 nationality | -0.277*** |
|  | (0.027) |
| Age | -0.011*** |
|  | (0.002) |
| ***Ph.D. curriculum characteristics*** |  |
| # of publications during Ph.D. | 0.083*** |
|  | (0.011) |
|  |  |
| Common Last Name | -0.072*** |
|  | 0.016 |
| Other Controls | ✓ |
| Pseudo R2 | 0.11 |
| N obs | 3,351 |

Coefficients are average marginal effects. Standard errors clustered around students from the same university-research field and graduation-year are in parentheses. *** p<0.01. Controls include labor market characteristics at graduation, university-research field fixed effects, and graduation-year fixed effects.
