# Supplementary material for: Science and Engineering Ph.D. Students’ Career Outcomes, by Gender
Source: PLoS One. 2015 Aug 5;10(8):e0133177. doi: 10.1371/journal.pone.0133177 (PMC4526637; doi:10.1371/journal.pone.0133177)
Supplement: S4 Table — Standard errors clustered around students from the same university-research field and graduation-year are in parentheses. ** p<0.05, *** p<0.01. Controls include Ph.D. demographic and predetermined characteristics, Ph.D. number of publications and involvement in applied projects, supervisor characteristics, labor market characteristics at graduation, university-research field fixed effects, and graduation-year fixed effects. We report coefficient estimates with and without the Mills’ ratio. (DOCX) [file pone.0133177.s004.docx]

**S4 Table. Linear probability models for being employed in academia, industry, and public administration (with Mills’ ratios included)**

|  | Probability of being employed in academia | | Probability of being employed in industry | | Probability of being employed in public administration | |
| --- | --- | --- | --- | --- | --- | --- |
|  |  |  |  |  |  |  |
| Female | 0.071*** | 0.058** | -0.108*** | -0.095*** | 0.037*** | 0.037** |
|  | (0.021) | (0.023) | (0.019) | (0.020) | (0.014) | (0.016) |
| Mills' ratio |  | 0.180 |  | -0.183 |  | 0.003 |
|  |  | (0.165) |  | (0.154) |  | (0.088) |
| Other Controls | ✓ | ✓ | ✓ | ✓ | ✓ | ✓ |
| R2 | 0.15 | 0.15 | 0.15 | 0.15 | 0.03 | 0.03 |
| N obs | 2,345 | 2,345 | 2,345 | 2,345 | 2,345 | 2,345 |

Standard errors clustered around students from the same university-research field and graduation-year are in parentheses. ** p<0.05, *** p<0.01. Controls include Ph.D. demographic and predetermined characteristics, Ph.D. number of publications and involvement in applied projects, supervisor characteristics, labor market characteristics at graduation, university-research field fixed effects, and graduation-year fixed effects. We report coefficient estimates with and without the Mills’ ratio.
